# Pharmacokinetic Assessment of Drug Efflux from Erythrocytes Loaded with Corticosteroid Esters

**DOI:** 10.1007/s11095-025-03913-4

**Published:** 2025-08-15

**Authors:** William J. Jusko, Ruihong Yu

**Affiliations:** Department of Pharmaceutical Sciences, Division of Pharmacokinetics, Pharmacodynamics, and Systems Pharmacology, School of Pharmacy and Pharmaceutical Sciences, State University of New York at Buffalo, 404 Pharmacy Building, Buffalo, NY 14214 USA

**Keywords:** betamethasone, dexamethasone, mathematical modeling, pharmacokinetics, red blood cells

## Abstract

**Purpose:**

Drugs can be found in erythrocytes (RBC) in accordance with their physicochemical and specific binding properties, but particularly high concentrations can be attained using an ex vivo hypotonic pre-swelling method. Pharmacokinetic (PK) methodology and characterization of *in vivo* data for RBC-loaded corticosteroid prodrugs was sought.

**Methods:**

Three studies providing in vitro and *in vivo* assessments of the pharmacokinetics of steroid ester prodrugs loaded into RBC were found. A PK model involving three fractional input rates and two-compartment disposition was applied.

**Results:**

After their sodium phosphate ester pro-drugs were loaded into RBC and dosed intravenously, dexamethasone (DEX) and betamethasone (BET) plasma concentrations were markedly prolonged with three phases in humans and animals. For DEX in humans, a PK model accounted for the typical biexponential disposition of the active steroid and for input of a large fraction (0.72) released within 1 h, a small fraction (0.27) released over several hours (*t*_*1/2*_ = 5.5 h), and a very small fraction (0.008) released extremely slowly (*t*_*1/2*_ = 109 h). The DEX RBC concentrations were expected to remain far higher than plasma concentrations for this prolonged time. The PK model was also applied to DEX in rabbits and BET in rats with generally similar results and indicating full bioavailability.

**Conclusions:**

The proposed PK methodology well characterized the input properties of RBC-loaded controlled-release formulations of corticosteroids in animal and humans in context of the flip-flop kinetics of the released drug. The model may be relevant to other types of RBC-loaded therapeutic agents.

**Graphical Abstract:**

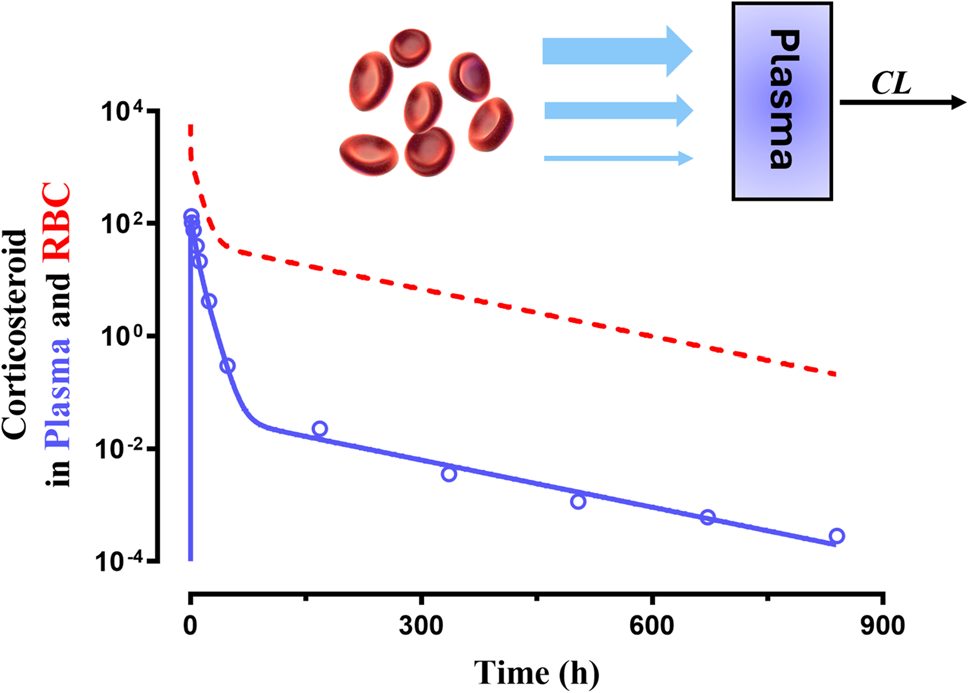

## Introduction

There is considerable interest in using either loaded or modified erythrocytes as carriers for extended *in vivo* delivery of drugs and other therapeutic agents. Several comprehensive reviews have summarized the extensive efforts in this field [1–4]. They provide descriptions of the physicochemical and physiological factors that impact RBC drug formulation and delivery, but applications of quantitative pharmacokinetic (PK) methodology for *in vivo* data seems to be lacking.

Glucocorticoids (GCs) such as dexamethasone (DEX) and betamethasone (BET) are in a class of drugs often used as anti-inflammatory and immunosuppressive agents. They are commonly given by various routes of administration as either their free alcohol forms or as the more soluble pro-drugs such as the 5’-sodium phosphate esters. Poorly soluble esters such as BET acetate are dosed either intramuscularly (IM) or locally to provide slow dissolution and gradual conversion to the active form [5]. The loading of DEX sodium phosphate (DSP) or BET sodium phosphate (BSP) into erythrocytes using an ex vivo hypotonic pre-swelling method offers promise for extended delivery of these agents [2, 3, 6]. The DEX-RBC formulation, in particular, is undergoing clinical trials for treating children with ataxia telangiectasia [7] and appears to be the only formulation with available PK data in humans.

BET is an epimer of DEX with similarities in their physiochemistry and PK properties. The molecular weight of both drugs is 392.5 g/mol, with moderate lipophilicity (BET log P = 1.14, DEX = 1.83) and water solubility (BET 66.5 mg/L, DEX 89 mg/L) (Drugbank Database). BET has a larger volume of distribution (*V*_*d*_) and smaller clearance (*CL)* compared to DEX, producing a longer half-life $\left({t}_{1/2\beta }=10.2\text{ vs }5.2\text{ h}\right)$ in humans [5, 8]. Their commercial formulations as DSP and BSP allow use of more concentrated solutions for IV injection and the phosphate ester hydrolyzes rapidly in blood. The attainable high concentration and ionized state facilitate their incorporation into and retention in RBC, wherein they are subsequently converted *in vivo* to the free alcohol forms by acid phosphatase [9]. In turn, as will be shown, such pro-drug loaded RBC release and produce sustained *in vivo* delivery of the active steroids.

The presence of drugs in RBC has been considered a “neglected compartment” in pharmacokinetics (PK) and pharmacodynamics (PD) [10]. Operation of physiologically-based PK models often utilizes whole blood:plasma ratios (*R*_*b*_) but assumes that drug concentrations in these phases are proportional and in rapid equilibrium. There is rarely knowledge and quantitation of drug efflux rates from RBC for inclusion in a PK model as was done for tacrolimus [11]. The recent comprehensive reviews describing drug delivery using carrier RBC describe the potential for applying PK models [2, 3]. It needs to be recognized, however, that a full PK characterization of controlled-delivery drugs with the occurrence of “flip-flop” PK requires joint assessment with its PK from IV or immediate-release dosage forms [12].

This report demonstrates basic approaches that may be generally useful for quantitating the PK of drugs after their RBC loading. The efflux properties of DEX and BET from loaded RBC will be shown as comprising three fractions and three rates lending insight into their observed *in vivo* plasma and RBC concentrations and therapeutic potential.

## Materials and Methods

### Data Collection

DEX and BET plasma concentration versus time data were obtained using Gsys2.4 (https://www.jcprg.org/gsys/) from published studies [6, 13, 14]. Published PK parameters for DEX in humans [8], rabbits [15] and BET in rats [16] were also utilized.

### Pharmacokinetic Model

**Fig. 1:**
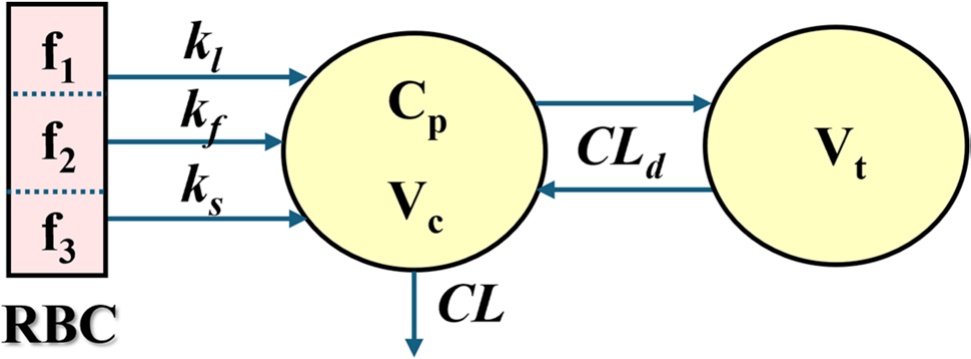
Pharmacokinetic model (FI/2CM) for the three-phase release of pro-drugs from loaded erythrocytes and the typical disposition behavior of the active steroids. The three fractions (*f*_*i*_) of the IV dose have individual rate constants (*k*_*i*_) for release from RBC and appearance in plasma. A two-compartment model (2CM) reflects the typical PK of IV-dosed drug in solution. Symbols are defined in the text and Table I.

The PK model structure is shown in Fig. 1 with three fractional inputs and two-compartment (FI/2CM) disposition. The equations used to assess the data from RBC-loaded and IV bolus drug PK were:

1 $${A}_{l}={Dose\cdot f}_{1}{\cdot e}^{-{k}_{l}\cdot t}$$

2 $${A}_{f}={Dose\cdot f}_{2}{\cdot e}^{-{k}_{f}\cdot t}$$

3 $${A}_{s}={Dose\cdot f}_{3}{\cdot e}^{-{k}_{s}\cdot t}$$

4 $$\frac{d{C}_{p}}{dt}=\left[{k}_{l}\cdot {A}_{l}+{k}_{f}\cdot {A}_{f}+{k}_{s}\cdot {A}_{s}-CL\cdot {C}_{p}- {CL}_{d}\cdot \left({C}_{p}-{C}_{t}\right)\right]/{V}_{c}$$

5 $$\frac{d{C}_{t}}{dt}={CL}_{d}\cdot \left({C}_{p}-{C}_{t}\right)/{V}_{t}$$

6 $${C}_{RBC}= \frac{Dose}{{V}_{RBC}}\cdot \left({f}_{1}{\cdot e}^{-{k}_{l}\cdot t}+{f}_{2}{\cdot e}^{-{k}_{f}\cdot t}+{f}_{3}{\cdot e}^{-{k}_{s}\cdot t}\right)$$

where ${C}_{p}$ is the DEX or BET plasma concentration in volume (${V}_{c}$), ${C}_{t}$ is the concentration in (${V}_{t}$), and *CL*_*d*_ and *CL* are distribution and elimination clearances. The initial conditions for *V*_*c*_ and *V*_*t*_ concentrations were zero for the RBC-loaded drug and *Dose/Vc* for the IV bolus drug. The efflux of drug from the *Dose* in the loaded RBC were modeled as Amounts (*A*_*i*_) in three fractional phases: *f*_*1*_, *f*_*2*_, and *f*_*3*_ with corresponding first-order rate constants *k*_*l*_, *k*_*f*_, and *k*_*s*_. The *C*_*RBC*_ is drug concentration in red blood cells, and *V*_*RBC*_ is either the *in vitro* volume of the RBC formulation (e.g., HCT·50 mL in humans) or the total *in vivo* RBC volume. The PK were adjusted for the fact that the actual DEX or BET in plasma comprises 76% of the stated doses of the pro-drugs. It was assumed that bioavailability (*F*) of the drug-loaded RBC was 100% and thus the *CL* applied to all data for each formulation.

### Additional Modeling

The human PK data for DEX were also fitted to the poly-exponential equation:

7 $$\begin{array}{cc}{C}_{p}={Dose\cdot C}_{1}{\cdot e}^{-\lambda 1\cdot t}+{Dose\cdot C}_{2}{\cdot e}^{-\lambda 2\cdot t}& -\end{array}Dose\cdot \left({C}_{1}+{C}_{2}\right){\cdot e}^{- ka\cdot t}$$

where *C*_*1*_ and *C*_*2*_ are intercepts, *λ*_*1*_ and *λ*_*2*_ are slopes, and *k*_*a*_ is an input rate constant. Apparent clearance (*CL/F*) can be calculated from the Dose/AUC as:

8 $$CL/F=Dose/\left(\frac{C_1}{\lambda_1}+\frac{C_2}{\lambda_2}-\frac{C_1+C_2}{k_a}\right)$$

### Model Fitting

The maximum likelihood method in ADAPT 5 was used in the nonlinear regression [17]. The variance model was ${Var}_{i}={({\sigma }_{1}+{\sigma }_{2}\bullet {Y}_{i})}^{2}$ where ${Var}_{i}$ presents the variance of the *i*th data point, ${Y}_{i}$ is the *i*th model prediction, and ${\sigma }_{1}$ and ${\sigma }_{2}$ are the variance model parameters that were estimated together with the system parameters. The models were evaluated by the goodness-of-fittings, visualization inspection, Akaike Information Criterion (AIC), and Coefficient of Variation (CV%) of the estimated parameters. All figures were generated by GraphPad Prism 9.1 (GraphPad, San Diego, CA).

## Results

**Fig. 2:**
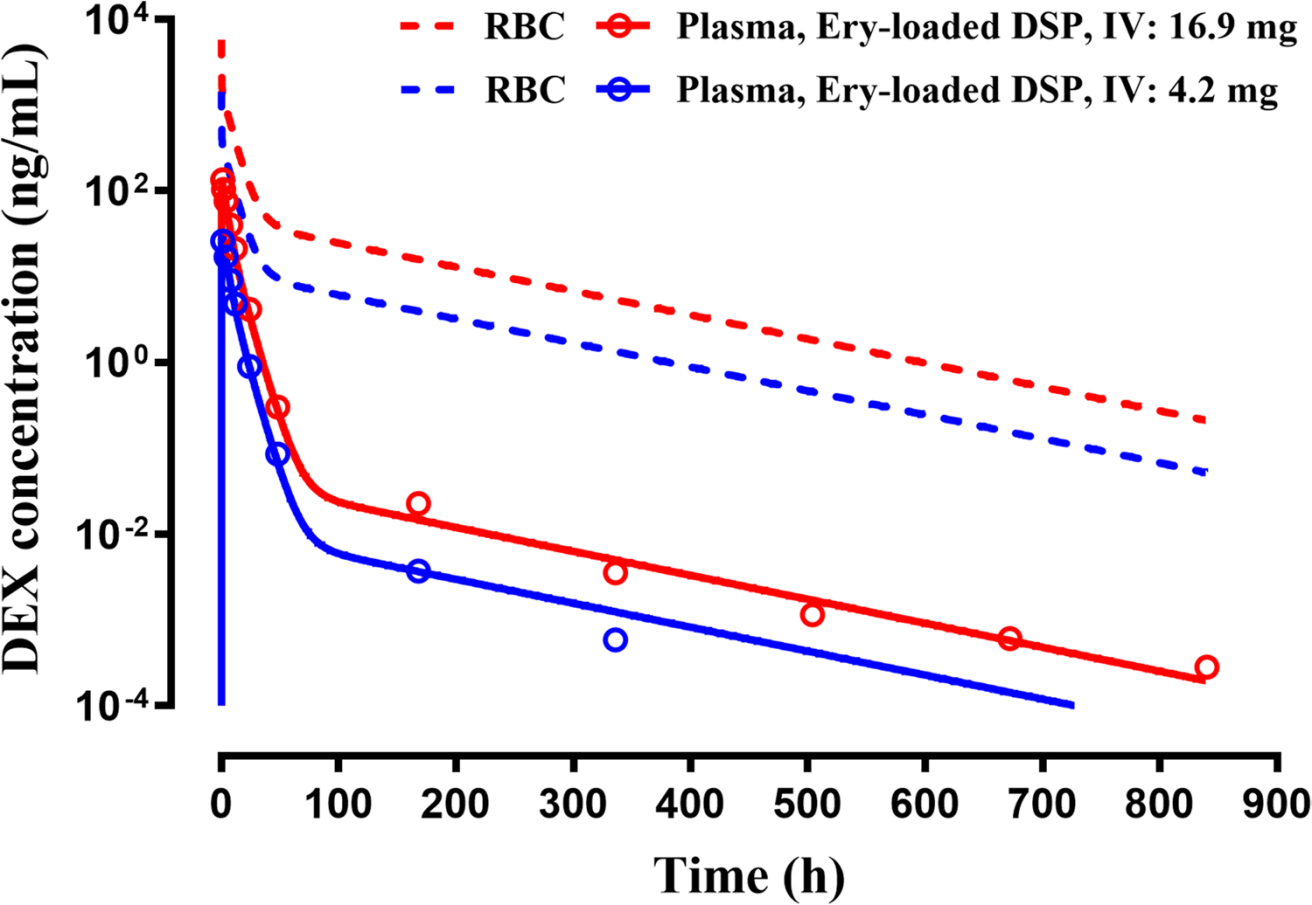
The time-course of measured plasma (solid lines) and expected RBC concentrations (broken lines) of dexamethasone (DEX) after IV administration of the indicated doses of dexamethasone sodium phosphate (DSP) in RBC in human subjects. Symbols are drug concentrations and curves are joint model fittings or predictions. Data were digitized from [6].

The mean plasma concentrations of DEX after IV administration of loaded RBC containing DSP in healthy human subjects as published by [6] are shown in Fig. 2. The PK shows an early high concentration with a *C*_*max*_ of 135 ng/mL for the 16.9 mg dose and 26 ng/mL for the 4.2 mg dose, both at 1.1 h (*t*_*max*_) after the infusions. The plasma concentrations decline over the next 90 to 100 h and then very slowly over the remaining 700 h. The PK of an IV dose with a half-life of 5 h would drop quickly within 24 h [5] thus indicating that flip-flop kinetics pertain and that slow input mechanisms were present for RBC-loaded DEX. The fittings with the proposed model (Fig. 1) captured the DEX data for both doses extremely well. The PK parameters listed in Table I show very low CV% values. As it was not possible to distinguish any data points prior to the *t*_*max*_ at 1.1 h (as listed in the publication), it was necessary to optimize the first rate constant (*k*_*l*_) to capture the *C*_*max*_ and *t*_*max*_. The FI/2CM model accounts for the input of a large fraction (0.72) released quickly over 1 h, a small fraction (0.27) released over many hours (*t*_*1/2*_ = 5.5 h), and a very small fraction (0.008) released extremely slowly (*t*_*1/2*_ = 109 h). The predicted RBC-DSP concentrations were generated based on Eq. 6 using a human RBC volume of 2.22 L. These expected measured concentrations were about 1071 times higher than and paralleled the DEX plasma concentrations.

**Table I:** The Pharmacokinetic Parameters (CV%) of Dexamethasone (DEX) in Humans and Rabbits and Betamethasone (BET) in Rats.

Parameter (units)	Definition	DEX in humans	DEX in rabbits	BET in rats
*f*_*1*_	Phase 1 fraction	0.7211 (11)	0.9038 (4.2)	0.2662 (42)
*f*_*2*_	Phase 2 fraction	0.2707 (30)	0.0371 (91)	0.307 (39)
*f*_*3*_	Phase 3 fraction	0.0082	0.0591	0.4268
*k*_*l*_ (h^−1^)	First-order rate constant of phase 1-unloading	4.3 (Fixed)	1.025 (Fixed)	8.1 (Fixed)
*k*_*f*_ (h^−1^)	First-order rate constant of phase 2-fast	0.1248 (11)	0.1355 (143)	0.04223 (51)
*k*_*s*_ (h^−1^)	First-order rate constant of phase 3-slow	0.006349 (5.3)	0.005016 (23)	0.002444 (71)
*CL* (mL/h/kg)	Elimination clearance	206 (6.6)	312(Fixed)	302 (Fixed)
*CL*_*d*_ (mL/h/kg)	Distribution clearance	9.27 (Fixed)	1135 (Fixed)	492 (23)
*V*_*c*_ (mL/kg)	Volume of central compartment	884.5(Fixed)	31.0 (Fixed)	54.0 (Fixed)
*V*_*t*_ (mL/kg)	Volume of peripheral compartment	87.2 (Fixed)	592 (Fixed)	626 (Fixed)

The Fixed Values reflect the 2CM parameters from published reports [8, 15, 16]

**Fig. 3:**
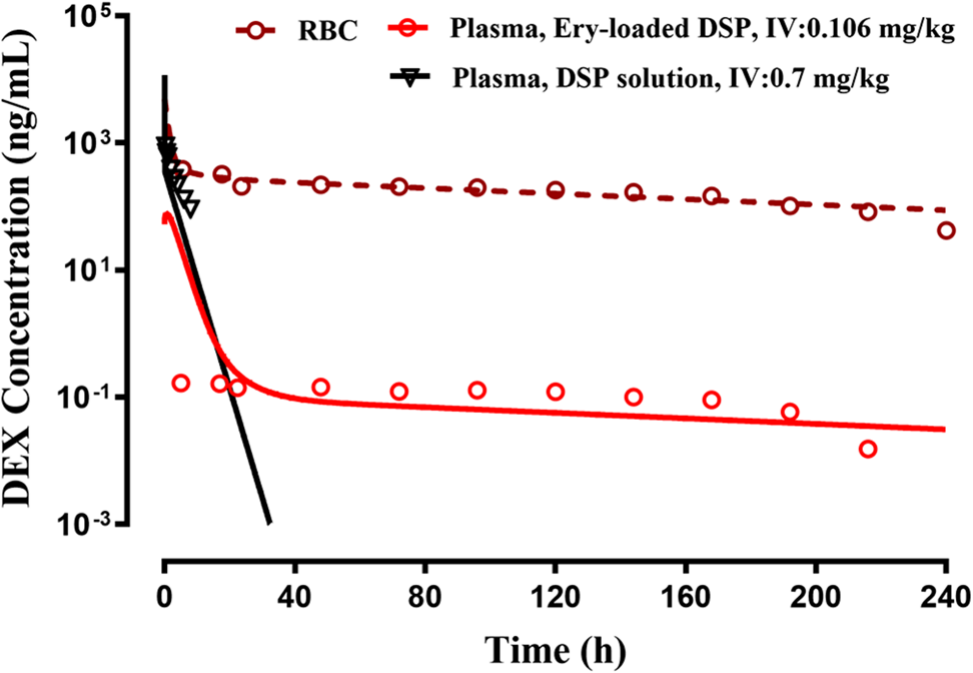
The time-course of measured dexamethasone (DEX) concentrations in plasma and RBC after IV administration of the indicated doses of RBC-loaded dexamethasone sodium phosphate (DSP) and a DSP solution in rabbits. Symbols are drug concentrations and curves are joint model fittings. Data were digitized from [14].

The plasma and red blood cell concentrations of DEX in rabbits dosed with RBC-loaded DSP and an IV dose of DSP in solution are shown in Fig. 3 [14]. In this case, the three input fractions advantageously jointly fitted loss of DEX from the RBC and its appearance in plasma. Equation 6 was operated with the assumption that the 3.0 kg rabbits had a blood RBC volume (*V*_*RBC*_) of 64.8 mL. The RBC and plasma concentrations again show three phases that were well-captured by the FI/2CM. The three fractions were similar to those in humans (Table I) with parameters having reasonable CV%. The IV dose of DSP in solution showed very rapid decline over the first day with the half-life of 3.6 h while the RBC-DSP concentrations were maintained at 2800 times the plasma concentrations. The DSP-loaded RBC was shown to hold the drug *in vitro* with a half-life of about 80 h confirming the *in vitro* stability of this formulation [14].

**Fig. 4:**
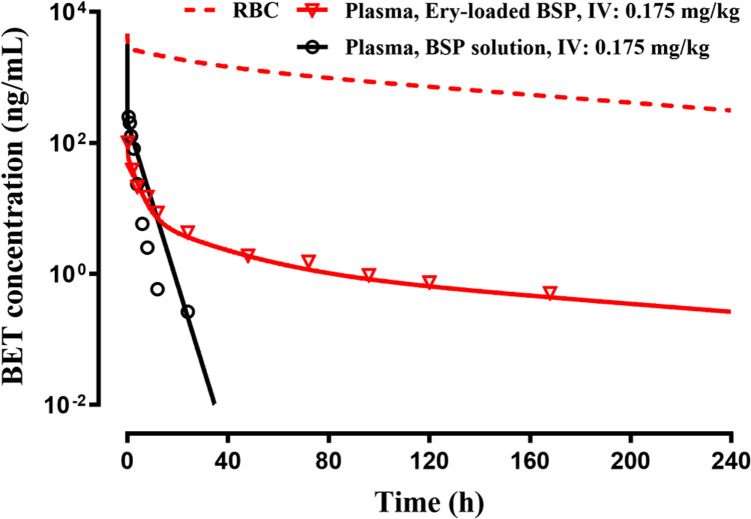
The time-course of measured plasma (solid lines) and expected RBC concentrations (broken line) of betamethasone (BET) after IV administration of the indicated doses of betamethasone sodium phosphate (BSP) in solution and in loaded RBC in rats. Symbols are drug concentrations and curves are joint model fittings or predictions. Data were digitized from [13].

The plasma concentrations of BET in rats after IV administration of loaded RBC containing BSP and BSP in solution as published by [13] are shown in Fig. 4. The PK shows an immediate high BET concentration. The plasma concentrations decline over the next 48 h (*t*_*1/2*_ = 16.5 h) and then very slowly (*t*_*1/2*_ = 100.0 h) over the remaining 144 h. The PK of the IV dose drops quickly within 24 h, confirming the flip-flop behavior with three-phase release of BET from the loaded RBC. The fittings with the proposed FI/2CM model (Fig. 1) captured the data for both doses quite well. The PK parameters listed in Table I show reasonable CV% values. The predicted RBC concentrations were generated based on Eq. 6 using a rat RBC volume of 5.7 mL and show very high concentrations that parallel the plasma DEX concentrations.

While the general appearance of the PK profiles were similar for DEX and BET, the fractional release properties of BET differed. BET modeling produced three fractions that were quite similar (0.2–0.4). Of course, all three were different studies, different conditions of loading the RBC, different species, and perhaps different hydrolysis rates of the phosphate esters. Never-the-less, the general PK model had the same structure for both drugs in the three species. Both formulations exhibited high bioavailability (essentially = 1) as might be expected for IV dosed drugs as the fittings assumed that the IV clearances pertained to both the RBC-loaded and IV doses of the drugs.

An alternative approach to characterizing the PK of drug-loaded RBC is fitting with a poly-exponential equation (Eq. 7). This produced essentially identical fittings of the DEX PK (graphical abstract and Fig. 2). Values obtained were: *C*_*1*_ and *C*_*2*_ = 6609 and 2.649 pg/mL per mg, *λ*_*1*_ and *λ*_*2*_ = 0.1288 and 0.006368 h^−1^, with fixed *k*_*a*_ = 4.3 h^−1^. This has the merit of providing half-life values for the three phases that are very similar to those from the three rate constants in the PK model and produces an apparent *CL* from *AUC* = 848.2 ng/mL/hr for the higher dose. However, poly-exponential fittings do not provide parameters of a 2CM model for RBC-loaded drug, as the occurrence of flip-flop PK produces intercept and slope values that will not reveal the true distribution properties as would be obtained after an IV bolus dose of the drug. Only the *CL*/*F* value is valid as the AUC applies to both approaches (Eq. 8).

The initial unloading phase of the PK profile in humans is supported by a calculation of the amount of DEX in the body at the *t*_*max*_ for the 16.9 mg dose of DSP. This provides *C*_*max*_ · *V*_*ss*_/(0.76 · Dose), where *C*_*max*_ is 135 (SD = 16) ng/mL [6] and *V*_*ss*_ is 72.44 L [8], reflecting 77% of the administered dose. This is a large fraction that is consistent with *f*_*1*_ (0.72) as some drug would have been eliminated prior to *t*_*max*_. Further, at the time of *C*_*max*_, it can be estimated (RBC concentration · RBC volume) that about 24% of the drug is retained in the RBC.

## Discussion

There are additional *in vivo* PK studies with steroid ester-loaded RBC in animals showing consistency with the three studies analyzed. Cortisol-21-phosphate was loaded into RBC of rats by hypotonic swelling, injected IV, and showed sustained concentrations in both collected RBC and plasma over 10 days [18]. Another study claimed to have loaded human RBC using a 1% solution of prednisolone and, after IV dosing in rats along with prednisolone in solution, showed higher tissue concentrations over 5 h with the RBC-loaded dose [19]. Prednisolone has an aqueous solubility of only 0.24 mg/mL or 0.024%, so this study is questionable unless they actually used the sodium phosphate prodrug.

The FI/2CM PK model employed was very basic and worked well in characterizing the available plasma data for DEX and BET released from the loaded RBC. Ordinarily, use of IV or immediate release PK data in deconvolution of the plasma profiles is helpful for resolving variable input kinetics of drugs [20], but the PK processes determining availability of DEX and BET in these studies are quite evident. The PK model offers the advantage of quantitating the apparent fractions and rates contributing to drug appearance in plasma. The application of the poly-exponential equations in fitting the three phases was supportive of the FI/2CM model and offers an alternative means of simulating multiple-dose expectations. These studies also indicate that animal models may be very useful for assessing the PK of drug-loaded RBC.

The loading procedure of the RBC with DSP in humans worked quite well *in vitro* with the loaded cells found to hold a large fraction (73–78%) of their initial content for 24 h *in vitro* after the loading and washing process [6]. Apparently the acid phosphatase that has been shown to hydrolyze DSP [9] is inactive *in vitro*. It has been stated that DSP is “slowly dephosphorylated by erythrocyte resident enzymes” [21]. This occurring *in vivo* may partly account for the second and third phases of DEX PK. Notably, considerable concentrations of the pro-drug esters are retained for a lengthy time in RBC as shown by our simulations (Figs. 2 and 4) and confirmed with direct measurements in rabbits (Fig. 3). Free DEX and BET are expected to equilibrate rapidly between RBC and plasma with their good lipophilicity and modest plasma protein binding (about 70–80%) [22], but it is likely that some DSP itself is released soon after *in vivo* infusion of the loaded cells. When DSP and BSP in solution are injected directly into blood, alkaline phosphatase enzymes rapidly hydrolyze the esters to the free alcohol forms [5, 23] and blood samples need stabilization with sodium arsenate to prevent this from happening after blood collections.

There may be additional factors contributing to the second (*t*_*1/2*_ = 5.5 h) and prolonged phases (*t*_*1/2*_ = 109 h). The recovery of chromium-labeled RBC after dosing in humans shows a very rapid loss of about 20% of the cells within 1 day and a slower loss phase with a half-life of about 40 days [6]. Thus, breakdown of the loaded RBC in these two phases may be contributing to the appearance and persistence of DEX in plasma. DEX primarily undergoes metabolism and excretion by the liver and kidneys [8], but the possibility exists that the RBC are also delivering drug to these organs and to macrophages [9]. The studies in rabbits following DEX in both RBC and plasma show 2800-fold higher DEX (actually DSP) concentrations in RBC during the terminal phase (Fig. 3) [14]. This is far higher than the usual blood:plasma ratio (*R*_*b*_) of 0.7 expected from a typical free drug equilibration process [22], indicating considerable retention of some DSP in the RBC according to the smaller fractions.

The pharmacological relevance of this persistence of very high RBC concentrations is intriguing. Senescence of RBC largely involves uptake by macrophages in the spleen and liver. Studies with liposomal methylprednisolone in rats has demonstrated enhanced drug uptake, prolonged receptor binding, and extended suppression of lymphocyte proliferation in these tissues [24]. The RBC-loaded with DSP have shown enhanced *in vitro* inhibition of lipopolysaccharide stimulated cytokine production in macrophages [9]. Thus RBC-DSP targeting of lymphocyte trafficking and activation in lymphoid tissues may partly account for its biological activity.

An additional mechanism may contribute to the long terminal phase concentrations of these steroids. Coker *et al.* [6] measured DEX concentrations for a longer time and to much lower concentrations (down to about 1 pg/mL) than had ever been done previously after DEX dosing in humans or animals. The possibility exists that the prolonged terminal phase is affected by or contributing to slow release of DEX from glucocorticoid receptors (GR) in tissues. The GR are found throughout mammalian tissues [25] and the DEX receptor binding (*K*_*d*_) is about 3.5 nM or 1400 pg/mL [26]. This plasma concentration was reached at about 50 h after the 16.9 mg dose of DSP (Fig. 2) but partial GR binding should continue thereafter. Prolonged blood and tissue sampling of prednisolone [27], methylprednisolone [28], and DEX (unpublished observations) in rats have revealed long terminal phases that were attributed partly to GR binding in tissues.

The loading dose/sustained PK profiles of DEX have offered promise in therapeutic use as DSP-loaded RBC have been studied in patients with COPD [21] and other conditions and clinical trials are underway with a commercial product, EryDex [7]. The RBC product is being dosed once monthly. The suppression of cortisol occurs with a DEX *IC50* of about 0.05 ng/mL or 50 pg/mL [29]. Thus, the DEX profile shown in Fig. 2 would indicate that, in a typical adult subject, DEX plasma concentrations would remain well below this value for much of a 30-day (720 h) dosing period. This should abrogate some of the typical chronic corticosteroid side effects.

This PK analysis utilized accessible PK profiles of corticosteroids relevant to RBC loading that could be found in the literature and demonstrates modeling concepts and approximate parameter values. This study has several limitations due to its assumptions and data sources. The DEX and BET studies in rabbits and rats provide companion IV PK data that directly demonstrate and verify its distribution and elimination properties, but DEX PK has been well studied in humans [5, 23]. Stabilization of plasma samples was not evident to prevent *in vitro* post-collection conversion of DSP or BSP if the phosphate esters were initially present in plasma after blood collection [5]. Further studies should confirm the expected high *in vivo* RBC concentrations of DSP in humans. The specificity and accuracy of the analytical assays are uncertain. Of course, by necessity, the data were digitized from mean concentration profiles that were assumed to best reflect the PK properties of the drug in each study. Lastly, future studies will determine whether adaptations of the present FI/2CM will be needed to handle the input and disposition kinetics of other therapeutic agents loaded into RBC.

## Conclusions

A relatively simple PK model (FI/2CM) that considers three phases of release of DEX and BET from loaded RBC along with jointly including the disposition kinetics of IV drug provided successful quantitation of published data for these drugs. This modeling approach may be useful for interpreting *in vitro* and *in vivo* data, provide the basis for simulations of multiple-dose regimens, and serve as the structural model in assessing population PK data from Phase I and II clinical trials.

## Data Availability

Data supporting the findings of this study are available in the cited publications and in the graphs of this article.

## References

[1] Muzykantov VR. Drug delivery by red blood cells: vascular carriers designed by mother nature. Expert Opin Drug Deliv. 2010;7(4):403–27. 20192900 10.1517/17425241003610633 PMC2844929

[2] Koleva L, Bovt E, Ataullakhanov F, Sinauridze E. Erythrocytes as carriers: from drug delivery to biosensors. Pharmaceutics. 2020. 10.3390/pharmaceutics12030276. 32197542 10.3390/pharmaceutics12030276 PMC7151026

[3] Glassman PM, Villa CH, Ukidve A, Zhao Z, Smith P, Mitragotri S, Russell AJ, Brenner JS, Muzykantov VR. Vascular drug delivery using carrier red blood cells: focus on RBC surface loading and pharmacokinetics. Pharmaceutics. 2020. 10.3390/pharmaceutics12050440. 32397513 10.3390/pharmaceutics12050440 PMC7284780

[4] Biagiotti S, Perla E, Magnani M. Drug transport by red blood cells. Front Physiol. 2023;14:1308632. 38148901 10.3389/fphys.2023.1308632 PMC10750411

[5] Jobe AH, Milad MA, Peppard T, Jusko WJ. Pharmacokinetics and pharmacodynamics of intramuscular and oral betamethasone and dexamethasone in reproductive age women in India. Clin Transl Sci. 2020;13(2):391–9. 31808984 10.1111/cts.12724 PMC7070803

[6] Coker SA, Szczepiorkowski ZM, Siegel AH, Ferrari A, Mambrini G, Anand R, Hartman RD, Benatti L, Dumont LJ. A study of the pharmacokinetic properties and the in vivo kinetics of erythrocytes loaded with dexamethasone sodium phosphate in healthy volunteers. Transfus Med Rev. 2018;32(2):102–10. 29031409 10.1016/j.tmrv.2017.09.001

[7] Zielen S, Crawford T, Benatti L, Magnani M, Kieslich M, Ryan M, Meyts I, Gulati S, Borgohain R, Yadav R, Pal P, Hegde A, Kumar S, Venkateswar A, Udani V, Vinayan KP, Nissenkorn A, Fazzi E, Leuzzi V, Stray-Pedersen A, Pietrucha B, Pascual SI, Gouider R, Koenig MK, Wu S, Perlman S, Thye D, Janhofer G, Horn B, Whitehouse W, Lederman H. Safety and efficacy of intra-erythrocyte dexamethasone sodium phosphate in children with ataxia telangiectasia (ATTeST): a multicentre, randomised, double-blind, placebo-controlled phase 3 trial. Lancet Neurol. 2024;23(9):871–82. 39152028 10.1016/S1474-4422(24)00220-5

[8] Krzyzanski W, Milad MA, Jobe AH, Peppard T, Bies RR, Jusko WJ. Population pharmacokinetic modeling of intramuscular and oral dexamethasone and betamethasone in Indian women. J Pharmacokinet Pharmacodyn. 2021;48(2):261–72. 33389521 10.1007/s10928-020-09730-z PMC7778726

[9] D’Ascenzo M, Antonelli A, Chiarantini L, Mancini U, Magnani M. Red blood cells as a glucocorticoids delivery system. In: Sprandel U, Way JL editors. Erythrocytes as drug carriers in medicine. Boston, MA: Springer; 1997. 10.1007/978-1-4899-0044-9_11.

[10] Hinderling PH. Red blood cells: a neglected compartment in pharmacokinetics and pharmacodynamics. Pharmacol Rev. 1997;49(3):279–95. 9311024

[11] Piekoszewski W, Chow FS, Jusko WJ. Disposition of tacrolimus (FK 506) in rabbits. role of red blood cell binding in hepatic clearance. Drug Metab Dispos. 1993;21(4):690–8. 7690698

[12] Yanez JA, Remsberg CM, Sayre CL, Forrest ML, Davies NM. Flip-flop pharmacokinetics–delivering a reversal of disposition: challenges and opportunities during drug development. Ther Deliv. 2011;2(5):643–72. 21837267 10.4155/tde.11.19 PMC3152312

[13] Zhang X, Qiu M, Guo P, Lian Y, Xu E, Su J. Autologous red blood cell delivery of betamethasone phosphate sodium for long anti-inflammation. Pharmaceutics. 2018. 10.3390/pharmaceutics10040286. 30567356 10.3390/pharmaceutics10040286 PMC6320894

[14] Ogiso T, Iwaki M, Ohtori A. Encapsulation of dexamethasone in rabbit erythrocytes, the disposition in circulation and anti-inflammatory effect. J Pharmacobiodyn. 1985;8(12):1032–40. 3834058 10.1248/bpb1978.8.1032

[15] Trenque T, Lamiable D, Vistelle R, Millart H, Leperre A, Choisy H. Comparative pharmacokinetics of two diastereoisomers dexamethasone and betamethasone in plasma and cerebrospinal fluid in rabbits. Fundam Clin Pharmacol. 1994;8(5):430–6. 7875637 10.1111/j.1472-8206.1994.tb00822.x

[16] Tamvakopoulos CS, Neugebauer JM, Donnelly M, Griffin PR. Analysis of betamethasone in rat plasma using automated solid-phase extraction coupled with liquid chromatography-tandem mass spectrometry. Determination of plasma concentrations in rat following oral and intravenous administration. J Chromatogr B Analyt Technol Biomed Life Sci. 2002;776(2):161–8. 12137997 10.1016/s1570-0232(02)00271-4

[17] D’Argenio D, Schumitzky A, Wang X. Adapt 5 User’s Guide: Pharmacokinetics/pharmacodynamic systems analysis software. BMSR: University of Southern California; 2009.

[18] Pitt E, Johnson CM, Lewis DA, Jenner DA, Offord RE. Encapsulation of drugs in intact erythrocytes: an intravenous delivery system. Biochem Pharmacol. 1983;32(22):3359–68. 6651861 10.1016/0006-2952(83)90363-5

[19] Shavi GV, Doijad RC, Deshpande PB, Manvi FV, Meka SR, Udupa N, Omprakash R, Dhirendra K. Erythrocytes as carrier for prednisolone: in vitro and in vivo evaluation. Pak J Pharm Sci. 2010;23(2):194–200. 20363699

[20] Ferron GM, Rochdi M, Jusko WJ, Scherrmann JM. Oral absorption characteristics and pharmacokinetics of colchicine in healthy volunteers after single and multiple doses. J Clin Pharmacol. 1996;36(10):874–83. 8930773 10.1002/j.1552-4604.1996.tb04753.x

[21] Rossi L, Serafini S, Cenerini L, Picardi F, Bigi L, Panzani I, Magnani M. Erythrocyte-mediated delivery of dexamethasone in patients with chronic obstructive pulmonary disease. Biotechnol Appl Biochem. 2001;33(2):85–9. 11277860 10.1042/ba20000087

[22] Song D, Sun L, DuBois DC, Almon RR, Meng S, Jusko WJ. Physiologically based pharmacokinetics of dexamethasone in rats. Drug Metab Dispos. 2020;48(9):811–8. 32601175 10.1124/dmd.120.091017 PMC7448200

[23] Song D, Jusko WJ. Across-species meta-analysis of dexamethasone pharmacokinetics utilizing allometric and scaling modeling approaches. Biopharm Drug Dispos. 2021;42(5):191–203. 33638217 10.1002/bdd.2266 PMC8859844

[24] Mishina EV, Jusko WJ. Inhibition of rat splenocyte proliferation with methylprednisolone: in vivo effect of liposomal formulation. Pharm Res. 1994;11(6):848–54. 7937524 10.1023/a:1018929824798

[25] Ballard PL, Baxter JD, Higgins SJ, Rousseau GG, Tomkins GM. General presence of glucocorticoid receptors in mammalian tissues. Endocrinology. 1974;94(4):998–1002. 4362047 10.1210/endo-94-4-998

[26] Nakada MT, Stadel JM, Poksay KS, Crooke ST. Glucocorticoid regulation of beta-adrenergic receptors in 3T3-L1 preadipocytes. Mol Pharmacol. 1987;31(4):377–84. 3033466

[27] Li X, DuBois DC, Almon RR, Jusko WJ. Physiologically based pharmacokinetic modeling involving nonlinear plasma and tissue binding: application to prednisolone and prednisone in rats. J Pharmacol Exp Ther. 2020;375(2):385–96. 32883831 10.1124/jpet.120.000191 PMC7604337

[28] Yu R, Jusko WJ. Physiologically based pharmacokinetic modeling: the reversible metabolism and tissue-specific partitioning of methylprednisolone and methylprednisone in rats. Drug Metab Dispos. 2024;52(7):662–72. 38653502 10.1124/dmd.124.001711 PMC11185821

[29] Krzyzanski W, Milad MA, Jobe AH, Peppard T, Bies RR, Jusko WJ. Population pharmacodynamic modeling of intramuscular and oral dexamethasone and betamethasone effects on six biomarkers with circadian complexities in Indian women. J Pharmacokinet Pharmacodyn. 2021;48(3):411–38. 33954911 10.1007/s10928-021-09755-y PMC8099395

